# Bacterial pathogens under high-tension: *Staphylococcus aureus* adhesion to von Willebrand factor is activated by force

**DOI:** 10.15698/mic2019.07.684

**Published:** 2019-06-11

**Authors:** Felipe Viela, Pietro Speziale, Giampiero Pietrocola, Yves F. Dufrêne

**Affiliations:** 1 Institute of Life Sciences, Université catholique de Louvain, Croix du Sud, 4-5, bte L7.07.06, B-1348 Louvain-la-Neuve, Belgium.; 2 Department of Molecular Medicine, Unit of Biochemistry, University of Pavia, Viale Taramelli 3/b, 27100 Pavia, Italy.; 3 Department of Industrial and Information Engineering, University of Pavia, Italy.; 4 Walloon Excellence in Life sciences and Biotechnology (WELBIO), Belgium.

## Abstract

Attachment of *Staphylococcus aureus* to platelets and endothelial cells involves binding of bacterial cell surface protein A (SpA) to the large plasma glycoprotein von Willebrand factor (vWF). SpA-mediated bacterial adhesion to vWF is controlled by fluid shear stress, yet little is currently known about the underlying molecular mechanism. In a recent publication, we showed that the SpA-vWF interaction is tightly regulated by mechanical force. By means of single-molecule pulling experiments, we found that the SpA-vWF bond is extremely strong, being able to resist forces which largely outperform the strength of typical receptor-ligand bonds. In line with flow experiments, strong adhesion is activated by mechanical tension. These results suggest that force induces conformational changes in the vWF molecule, from a globular to an extended state, leading to the exposure of cryptic binding sites to which SpA strongly binds. This force-sensitive mechanism may largely contribute to help *S. aureus* bacteria to resist shear stress of flowing blood during infection.

*Staphylococcus aureus* is a leading cause of endovascular diseases, such as infective endocarditis or heart valve prosthetic infection. During endovascular infection, the pathogen needs to attach to the endothelium and to resist shear stress of flowing blood. For that purpose, *S. aureus* expresses a repertoire of surface associated proteins (adhesins) that mediate bacterial attachment to extracellular matrix and endothelial cell surface components. A prototype of such interaction is the binding of protein A (SpA) to von Willebrand factor (vWF), a glycoprotein found in the vascular basement membrane and in the plasma. The mature vWF monomer consists of 2,050 residues and contains a number of domains, each involved in binding specific ligands, including FVIII, GPIbα and collagen. Endothelial cells and megakaryocytes are the only cells that synthesize vWF. vWF monomers are organized in large compacted multimers stored in organelles called Weibel-Palade bodies, which can be secreted upon extracellular stimuli.

SpA-dependent *S. aureus* adhesion to vWF is influenced by fluid shear, but the molecular details behind this interaction remain mysterious. This prompted us to investigate the strength and dynamics of the SpA-vWF bond using single-molecule atomic force microscopy. We discovered that SpA-dependent bacterial adhesion to vWF involves specific molecular bonds that are much stronger (∼2,000 piconewtons, pN) than most receptor-ligand interactions studied to date (∼200 pN). Remarkably, we found that the SpA-vWF bond is tightly mechanoregulated, being weak at low tensile force, but extremely strong at high force, thereby explaining why in high shear flow conditions bacteria adhere in large amounts to vWF surfaces (**Figure 1**).

**Figure 1:**
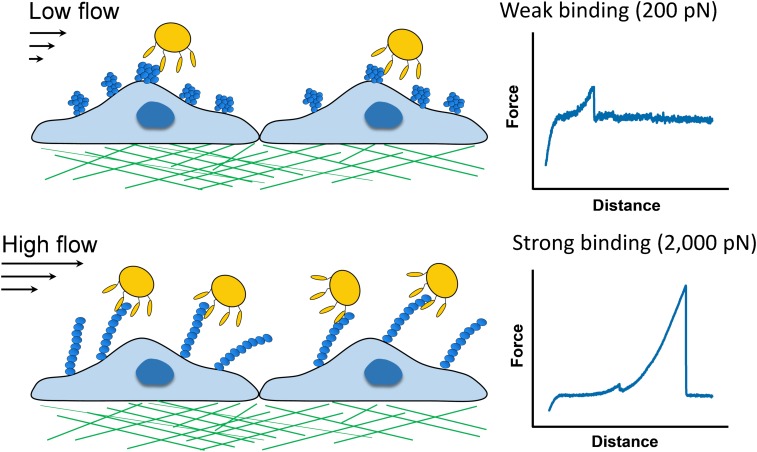
FIGURE 1: Force-induced activation of the SpA-vWF bond. Single-molecule pulling experiments led us to propose a mechanism whereby force-induced extension of vWF leads to the exposure of a cryptic binding site to which SpA proteins on the bacterial cell surface strongly bind. SpA-vWF bonds at high stress are much stronger (binding strength of ∼2,000 pN) than most receptor-ligand bonds measured to date, thus highlighting the importance of protein mechanobiology in bacterial adhesion.

Extremely strong forces (∼2,000 pN) were recently reported for the other staphylococcal adhesins SdrG, ClfA and ClfB, which bind to their ligands (e.g. fibrinogen) through the dock, lock, and latch (DLL) mechanism involving conformational changes in the adhesins that greatly stabilize the complex. Single-molecule experiments and steered molecular dynamics simulations have shown that the high mechanical stability of the SdrG-fibrinogen DLL complex results from a hydrogen bond network between the ligand peptide backbone and the adhesin. Our findings are novel and unexpected since SpA is structurally and functionally very different from other adhesins investigated so far, and does not involve DLL binding. We therefore believe that the force activation of the SpA-vWF bond primarily involves conformational changes in vWF rather than in the adhesin. Supporting this view, vWF is a mechanosensitive protein capable to respond to external forces, such as hydrodynamic shear in flowing blood. Under force, the vWF molecule transitions from a globular state to an extended chain conformation with exposure of intra-molecular globular domains. We speculate that force-induced extension of vWF may lead to the exposure of a cryptic high-affinity binding site to which SpA strongly binds. In addition, we cannot exclude that force-induced structural changes in the adhesin domains also contribute to the formation of such strong bonds. In the future, new structural biology data on the SpA-vWF complex should allow to clarify the molecular nature of this interaction.

Our study contributes to a growing body of literature showing that physical stress can profoundly impact bacterial behaviors. Specifically, there is increasing evidence that the adhesion of bacterial pathogens during infection can be strongly enhanced by fluid flow conditions. A prototypical example is the attachment of *E. coli* to epithelial cells, involving binding of FimH adhesin to mannose residues. This is achieved through so-called “catch bonds” that are strengthened by mechanical stress. Several studies have suggested that staphylococcal SdrG, ClfA and ClfB may use catch bond mechanisms to resist high shear stress during infections. These experiments suggest that in flow conditions the binding strength of bacterial proteins, measured under force at non-equilibrium, are more relevant than bulk affinity values obtained at equilibrium.

In conclusion, the mechanoregulated SpA-vWF bond rationalizes at the molecular level the ability of *S. aureus* to withstand high shear forces of flowing blood during endovascular infections (**Figure 1**). The force-activated bond might represent an interesting target for anti-staphylococcal therapy. Blocking of the SpA-vWF interaction is potentially important as entry of *S. aureus* into circulation can cause life-threatening vascular diseases. *S. aureus* adhesion to endothelial cells is a critical step in the destruction of cardiac valves with subsequent endocarditis and systemic dysfunction in the case of sepsis. Currently, treatment of blood stream infections involves aggressive antibiotic therapy for long periods of time. The greater the duration of exposure of an antibiotic to bacteria, the greater the risk is of developing antibiotic resistance. So, there is great interest in developing new strategies focusing on the prevention of bacterial attachment to the endothelium.

